# A Case of Uræmia Complicating Labor

**Published:** 1886-02

**Authors:** P. W. Turner

**Affiliations:** Abeline, Texas


### A CASE OF URÆMIA, COMPLICATING LABOR.

*By P. W. Turner, M. D., Abeline, Texas.*

(For Daniel's Texas Medical Journal.)

March 10th, 1884, Mrs. W., aged 20, was in labor with second child. I was summoned about 9 a. m. A midwife had been in attendance since the preceeding evening. Patient was slightly comatose, and no reliance was to be placed in subjective symptoms; temperature 105 degrees; pulse very rapid and feble; face was not flushed, neither was there any throbbing of carotid or temporal arteries. The symptoms more nearly resembled those of pernicious fever, but diligent enquiry into her former history led me to believe she was not the subject of a malarial attack. No œdema was perceptible, and being misinformed by one of the attendants that she had passed urine freely, and that the bowels were in a soluble condition, I was doubtful as to the nature of the case for the present, and so informed her poeple, withholding my diagnosis till an opportune time.

Palpation disclosed enormous distension of the abdomen with extreme rigidity; no intermittent uterine contractions could be felt. Digital examination revealed a slightly dilated os, and genital passages in a favorable condition.

I directed a warm bath in which I kept her half an hour, taking care to note any micturation which might occur, but there was none. The bath seemed to restore her to consciousness, after which she called for some bread and milk, and sitting up in bed ate it with apparent relish. This was at twelve o'clock, and I took my departure, promising to return in one or two hours. By that time the os was dilating as well as could be wished, but I found a return of the coma. Resorted again to the bath with same precautions as before, and applied ice to the head, but this time it failed to relieve the cerebral symptoms. Sent for Dr. Cochran who at once perceived the gravity of the situation, and we decided on instrumental delivery without delay. The os now being well opened, the Doctor punctured the membranes, and an enormous quantity of liq. amnii came away, after which the position of the child could be made out with distinctness through the thin abdominal walls.

I then introduced a silver catheter when only *one drop of urine came.* To make doubly sure I then used Nilaton's catheter; same result. The cause of the coma was now very obvious. On questioning another attendant she told me that the patient had *not passed any urine since the day before.* Suppression of urine;—that explained all. The forceps, however, were carefully introduced, and a dead fœtus brought forth. After removal of secondines the uterus contracted firmly; the case was attended with no hemorrhage.

We administered remedies, directed to the elimination of urea, but the coma increased until four o'clock the following morning, when the grim monster claimed its victim.

This case clearly shows the propriety of putting a pregnant woman in the care of a physician several months prior to her accouchment, whose duty it would be to look after and combat any renal complications that might arise, and thereby possibly averting such finale. A life lost, and probably from this neglect. It would furthermore show the importance of investigating the condition of the kidneys, from time to time, by an occasional analysis of the urine.

